# Influence of irrigation regimes on competition indexes of winter and summer intercropping system under semi-arid regions of Pakistan

**DOI:** 10.1038/s41598-020-65195-7

**Published:** 2020-05-18

**Authors:** Amanullah, Shah Khalid, Farhan Khalil, Imranuddin

**Affiliations:** 1 Agronomy Department, The University of Agriculture Peshawar, Peshawar, Pakistan; 2 Livestock Research and Development Station, Surezai, Peshawar, Pakistan

**Keywords:** Drought, Environmental impact

## Abstract

An assessment of the competitive indexes in intercropping of different winter and summer based intercropping systems were studied, with the aim of increasing the productivity of these crops. Four winter crops, wheat (*Triticum aestivum* L.), barley (*Hordeum vulgare* L.), fababean (*Vicia faba*) and rapeseed (*Brassica napus*) and four summer crops, sorghum (*Sorghum bicolor* L.), pearl millet (*Pennisetum typhoidum* L.), pigeonpea (*Cajanus cajan* L.) and mungbean (*Vigna radiate* L.) were grown under two irrigation regimes with the pattern of two crops in each intercropping system, at Agronomy Research Farm, The University of Agriculture, Peshawar, Pakistan in both winter and summer season during 2015–16 to 2016–17. The results showed that higher grain yield (kg ha^−1^) were recorded under sole cropping than intercropping. Higher grain yield was recorded in sole cropping, for all four crops. All crops grown in intercropping produced comparatively higher grains head^−1^ and seeds pod^−1^ than sole crop except pigeonpea. Intercropping systems were performed different in term of competition indexes which determined land utilization efficiency. Competition indexes revealed that in winter season wheat intercropped with fababean showed highest advantages of intercropping in term of land equivalent ratio (30%), relative crowding co-efficient (60%), actual yield loss (60%), area time equivalent ratio (27%), land utilization efficiency (83%), intercropping advantages (1060), monetary advantage index (Pakistani rupees (PKR) 46456) and system productivity index (3684) while in summer sorghum/pearl millet intercropped with pigeonpea was the most dominant intercropping systems in term of relative  crowding co-Efficient (40%), actual yield loss (50%), land utilization efficiency (60%) intercropping advantages (1150) and system productivity index (1914). Aggressivity and competition ratio showed that cereals especially barley in winter and sorghum in summer season was highly competitive crops in the intercropping system. Most of the competition indexes values were higher for winter crops under limited irrigated condition while in case of summer crops intercropping indexes were higher under full irrigated condition. It was concluded that wheat intercropped with fababean, and sorghum/millet intercropped with mung bean was the most successful intercropping systems in winter and summer seasons, respectively under both irrigation regimes, for the semiarid region of Pakistan.

## Introduction

Intercropping is advances techniques in which two or more crops are grown on the same piece of land at the same time, to get maximum benefits of it on sustainable basis^1,2^. For sustainable food and feed production intercropping is very essential, especially in limited land resources^3^ and inputs resources^4^. Intercropping is important component of sustainable agriculture^5,6^ and used in may developed and developing countries as a sustainable practices^7^. It provides security against crop yield reduction^8^. Intercropping have many benefits on sustainable base i.e. improving crop yield and soil fertility^9^ and productivity^10^, control soil erosion^11^. Intercropping have more advantaged over monocropping in term of crop productivity^12,13^, it provides highest land return^7,14^ and land use efficacy^3^, by improving crop yield^15^. It is an environmental friendly practice by decreasing the use of chemical fertilizers and pesticides^1^. Intercropping of cereals with legumes has been popular in tropics^16,17^ and rain-fed areas of the world^18–21^ due to its advantages for soil conservation^22,23^, weed control^24,25^ lodging resistance, yield increase^26^, and legume root parasite infections control^27,28^.

Intercropping is an attractive and simple practice which improving crop yield by increasing total productivity of crop per unit area per unit time^29^. Additionally, intercropping reduced weed density and improving plant health by reducing disease incidence^30^. During intercropping designing crop nutrients uptake mechanism is very important as sharing the same soil and environmental resources^31,32^. In case of legumes and non-legumes intercropping system, atmospheric nitrogen can be fixed by rhizobia bacteria present in legume nodules while nonlegumes relay only on soil nitrogen^2,33,34^. Intercropping of legumes with cereal crops can improve crop yield and growth by the using same available resources, intercropping increase available crop productivity as compared to each sole cropping^2,33,35,36^ also reported intercropping advantages over monocropping.

Drought is a significant limiting factor for agricultural productivity and generally inhibits plant growth through reduced water absorption and nutrient uptake. Decreased water availability generally results in reduced growth and final yield in crop plants. However, plant species in a mixed cropping system may vary in their responses to growth under water stress because water availability is known to be spatially heterogeneous distributed in time and space^37,38^. The current challenge in agriculture is to produce more yields by utilizing less water, especially in regions with limited land and water resources^39^. Efficient irrigation systems require the selection of an appropriate method for the crop growth, adequate monitoring of the irrigation system and of water delivery and appropriate application rates depending on the growth stage of the crop. Irrigation requirements differ depending on the locations, soil types and cultural practices^40^.

To describe the efficiency of an intercropping system researchers have developed many mathematical formulas to calculate the intercropping possible advantages, and to describe the intra and inter specific competition among or between components crop of an intercropping system. Among these formulas land equivalent ratio (LER)^41^, aggressivity (A)^42^, competition ratio^43^, area time equivalent ratio (ATER), Relative crowding coefficient (RCC)^44^, actual yield loss^45^, intercropping advantages^45^ and land utilization efficiency (LUE) are the most important^19,46^. These mathematical expressions help the researchers to interprets, display and summaries their result from an intercropping system. The indexes can help to showed different aspects of competition in plant communities, including competitive effects, competition intensity and outcome of competition^20^.

The effect of different irrigation on competition indexes of intercropping is not fully explored so far. Therefore, the study being reported in this manuscript was envisaged and performed under different irrigation regimes (full and limited irrigation) for knowing its effect on four winter crops like wheat (*Triticum aestivum* L.), barley (*Hordeum vulgare* L.), fababean (*Vicia faba*) and Rapeseed (*Brassica napus*) as winter crops and four summer crops like sorghum (*Sorghum bicolor* L.), pearl millet (*Pennisetum typhoidum* L.), pigeonpea (*Cajanus cajan* L.) and mungbean (*Vigna radiate* L.) under the semiarid region of Peshawar, Pakistan, for two consecutive years. The aim of the study was to evaluate the effect of irrigation regimes on different competition indexes of winter and summer intercropping system.

## Materials and Methods

### Field experiment

A two years field experiment was conducted during 2015–16 and 2016–17 at the Agronomy Research Farm, University of Agriculture, Peshawar. The experimental site has continental climate and is located at 34°27′12.46″N latitude and 71°27′56.4″E longitude with altitude of 359 m above sea level. Two adjacent fields were used separated by one meter viz. one under limited irrigation and the second one under full irrigation, both fields had similar physiochemical properties. The experiment under each irrigation regimes was conducted in randomized complete block design (combined over irrigation) having four replications. A sub plot size of 4 m × 4 m was used. Each plot was separate by 0.5 m earthen band to prevent the flow of water and mobile nutrients to nearby plots. A recommended rate basal dose of nitrogen and phosphorus for cereal is 120, 60 kg ha^−1^ while in case of legumes 30, 60 kg ha^−1^ N and P, were used, respectively. DAP was used as source of phosphorus and nitrogen, while the remaining nitrogen was applied through urea. In case of fababean, rapeseed, mungbean and pigeonpea all N (30 kg ha^−1^) was applied at sowing time, while for cereal crops nitrogen was applied in two equal splits (60 kg ha^−1^ at sowing time and 60 kg ha^−1^ at tillering stage). Phosphorus at the rate of 60 kg P ha^−1^ in the form of DAP was applied. Adjustment of N and P from DAP and urea were made. The required phosphorus was applied at the time of seedbed preparation. All other agronomic practices were kept normal and uniform for all the treatments. Physiochemical properties of the experimental site are given in Table 1. The treatments application and other standard agronomic practices detail are given in Table 2.

**Table 1:** Pre-sowing physiochemical properties of experimental site.

Property	Values/type
Sand (%)	17.23
Silt (%)	51.5
Clay (%)	31.23
Total Nitrogen (%)	0.04
Extractable Phosphorus (mg kg^−1^)	6.57
Extractable Zinc (mg kg^−1^)	0.7
Textural class	Silty clay loam
Organic Carbon	0.87%
soil pH	7.8

**Table 2:** Treatments application and other agronomic practices during both winter and summer season experiments.

Crops	Variety Name	Seed rate (kg ha)	Nitrogen (kg ha^−1^)	Phosphorus (kg ha^−1^)	Row to Row Distance (cm)
**Winter crops**					
Wheat	Atta Habib	120	120	60	25 cm
Barley	Bajaur Local	100	120	60	25 cm
Rapeseed	Abbasin-95	8	30	60	50 cm
Fababean	Local	150	30	60	50 cm
**Summer crops**					
Sorghum	Jowar-2011	75	120	60	50 cm
Pearl millet	Local	15	120	60	50 cm
Mungbean	Ramzan	30	30	60	50 cm
Pigeonpea	Local	40	30	60	50 cm
**Sole cropping of winter season**			**Sole cropping of summer season**		
Wheat	Twelve rows plot^−1^		Sorghum	Eight rows plot^−1^	
Barley	Twelve rows plot^−1^		Pearl millet	Eight rows plot^−1^	
Rapeseed	Six rows plot^−1^		Pigeonpea	Eight rows plot^−1^	
Fababean	Six rows plot^−1^		Mungbean	Eight rows plot^−1^	
**Intercropping of winter crops**				**Fertilizers Application**	**Time of application**
Wheat + Barley	Six rows of each crop were sown in alternate manner			Nitrogen	In two equal splits
Wheat + Fababean	Two rows of wheat with 01 row of Fababean in alternate manner			Phosphorus	In sowing time
Wheat + Rapeseed	Three rows of wheat with 01 row of rapeseed in alternate manner				
Fababean + Rapeseed	Three rows of fababean and rapeseed was grown in alternate manner				
**Intercropping of summer crops**				**Fertilizers Application**	**Time of application**
Sorghum + Pearl millet	Four rows of each crop were grown in alternate manner			Nitrogen (Urea)	Half at sowing and half after 1^st^ irrigation
Sorghum + Pigeonpea	Four rows of each crop were grown in alternate manner			Phosphorus (DAP)	In sowing time
Sorghum + Mungbean	Four rows of each crop were grown in alternate manner			Limited irrigation regime	Two irrigations
Pigeonpea + Mungbean	Four rows of each crop were grown in alternate manner			Full irrigation regime	Four irrigations

**Factor A. Irrigation**

1. **Limited irrigation**: only one irrigation (75 mm) was applied at booting stage of wheat to the winter crops, while in case of summer crops irrigations were given at pre-sowing and at anthesis stage of pearl millet.
2. **Full irrigation**: three irrigations, at tillering (95 mm), jointing (92 mm) and booting stage (75 mm) of wheat were applied to the winter crops, while in case of summer crops irrigation was applied at pre-sowing, stem elongation, anthesis and dough stage of pearl millet.

To calculate the amount of water applied at each irrigation “Float cut method” of Misra and Ahmad^47^ was applied.

**Experiment one: four winter crops (wheat, barley, rapeseed & fababean).**

**Factor B. Intercropping system (winter crops)**

1. Wheat sole crop
2. Barley sole crop
3. Fababean sole crop
4. Rapeseed sole crop
5. Wheat + barley
6. Wheat + fababean
7. Wheat + rapeseed
8. Barley + fababean
9. Barley + rapeseed
10. Fababean + rapeseed

**Experiment two: four summer crops (sorghum, pearl millet, mungbean & pigeonpea).**

**Intercropping system (Summer crops)**

1. Sorghum sole crop
2. Pearl millet sole crop
3. Mungbean sole crop
4. Pigeonpea sole crop
5. Sorghum + pearl millet
6. Sorghum + mungbean
7. Sorghum + pigeonpea
8. Pearl millet + mungbean
9. Pearl millet + pigeonpea
10. Mungbean + pigeonpea

### Data were recorded on the following parameters

#### Grains head^−1^ and seeds pod^−1^

Grains head^−1^ and seeds pod^−1^ were recorded by selecting five heads in cereals and ten pods in legumes in each treatments and grains were counted and then averaged.

#### Thousand grains/seeds weight (g)

For thousand grains/seeds weight data; after threshing, thousand grains were counted from each plot of each crop and weighed with the help of electronic balance.

#### Grain yield (kg ha^−1^)

The weighted harvested three central rows were sun dried, threshed, cleaned and weighed, and then weight were converted into kg ha^−1^ using the following formula.

$$Grain\,yield(kg\,h{a}^{-1})=\frac{Grain\,yield\,in\,three\,central\,rows\,in\,each\,plot}{(Row-row\,distance\times row\,length\times number\,of\,rows)}10000$$

### Aggressivity (A)

Aggressivity (A) indicates the relative yield increase in “a” crop is greater than of “b” crop in an intercropping system. The aggressivity can be derived from the following formula^42^:

$${\rm{A}}({\rm{main}}\,{\rm{crop}})=[{\rm{Yab}}/{\rm{Yaa}}]\,\mbox{--}\,[{\rm{Yba}}/{\rm{Ybb}}]$$

Similarly, Aggressivity of intercrops can also be calculated by the formula^42^:

$${\rm{A}}({\rm{intercrops}})=[{{\rm{Y}}}_{{\rm{ba}}}/{{\rm{Y}}}_{{\rm{bb}}}]-[{{\rm{Y}}}_{{\rm{ab}}}/{{\rm{Y}}}_{{\rm{aa}}}]$$

Where Y_ab_ is the yield of main crop in intercropping and Y_ba_ is the yield of intercrop crop and proportion of intercrop in intercropping.

### Competition ratio (CR)

The CR simply represents the ratio of individual land equivalent ratio (PLER) of the component crops and takes into account the proportion of the crops in which they were sown. In case of 1:1^43^

$$\begin{array}{c}{\rm{CR}}\,{\rm{main}}\,{\rm{crop}}=({\rm{PLER}}\,{\rm{main}}\,{\rm{crop}}/{\rm{PLER}}\,{\rm{intercrops}})\\ {\rm{CR}}\,{\rm{intercrops}}=({\rm{PLER}}\,{\rm{intercrops}}/{\rm{PLER}}\,{\rm{main}}\,{\rm{crop}})\end{array}$$

### Relative crowding coefficient (K)

The K is the measure of relative dominance of one species over the other in intercropping. For 1:1 pattern K was calculated as^44^:

$$\begin{array}{c}{\rm{K}}({\rm{system}})=[{\rm{k}}({\rm{main}}\,{\rm{crop}})]\times [{\rm{k}}({\rm{intercrop}})]\\ {\rm{k}}({\rm{intercrop}})=({\rm{Yba}})/({\rm{Ybb}}-{\rm{Yba}})\\ {\rm{k}}({\rm{main}}\,{\rm{crop}})=({\rm{Yab}})/({\rm{Yaa}}-{\rm{Yab}})\end{array}$$

Where Yab stand for grain yield of main crop in intercropping, Yba is the yield of intercrop in intercropping, Yaa is the yield of main crop in monocropping and Ybb is the yield of intercrop in monocropping. When the K value of the system is higher than one, there is a yield advantage, if the value of K is one there is no yield advantage and if less than one there is no yield advantage and the system has disadvantage^44^.

### Land equivalent ratios (LER)

The LER is the ratio of land required by pure (sole) crop to produce the same yield as that of intercrop. LER was determined according to the procedures used by Amanullah *et al*.^48^.

Equivalent Ratio (LER) was calculated by the following formula:

$$\begin{array}{c}{\rm{LER}}=[{{\rm{L}}}_{{\rm{a}}}+{{\rm{L}}}_{{\rm{b}}}]\\ {\rm{La}}=({{\rm{Y}}}_{{\rm{ab}}}/{{\rm{Y}}}_{{\rm{aa}}})\,{\rm{and}}\,{{\rm{L}}}_{{\rm{b}}}=({{\rm{Y}}}_{{\rm{ba}}}/{{\rm{Y}}}_{{\rm{bb}}})\end{array}$$

Where La and Lb stand for partial LERs for the component crops, Yab and Yba are the grain yield of component crop in intercropping and Yaa and Ybb are the grain yield of sole crop^43^.

### Actual yield loss (AYL)

The AYL is the proportionate yield loss or gain of intercrops in comparison to the corresponding sole crop. In addition, partial AYL (main crops) and AYL (intercrops) represent the proportionate yield loss or gain of each species in intercropping compared to their yield in sole crops. The negative or positive values of AYL indicate the advantage or disadvantage of the intercropping^21^. AYL was calculated by using the following formula^21^.

$$\begin{array}{c}{\rm{AYL}}({\rm{main}}\,{\rm{crop}})={[({\rm{Yab}}/{\rm{Zab}})/({\rm{Yaa}}/{\rm{Zaa}})]}^{-1};\\ {\rm{AYL}}({\rm{intercrop}})=[{({\rm{Yba}}/{\rm{Zba}}({\rm{Ybb}}/{\rm{Zbb}})]}^{-1},\\ {\rm{AYL}}({\rm{system}})={\rm{AYL}}\,({\rm{main}}\,{\rm{crop}})+{\rm{AYL}}\,({\rm{intercrop}})\end{array}$$

Where Yab, Zaa and Yba, Zbb, stands for grain yield of main crop and the ratio in which it was sown and the grain yield of intercrop and the ratio in which was sown in intercropping, respectively^21^.

### Area time equivalent ratio (ATER)

ATER provides more realistic comparison of the yield advantage of intercropping over monocropping in terms of time taken by component crops in the intercropping systems. ATER was calculated using the following formula^43^:

$$\begin{array}{rcl}{\rm{ATER}} & = & ({\rm{ATER}}({\rm{main}}\,{\rm{crop}})+{\rm{ATER}}({\rm{inter}}\,{\rm{crop}});\\ {\rm{ATER}}\,({\rm{main}}\,{\rm{crop}}) & = & {\rm{Y}}\,{\rm{main}}\,{\rm{crop}}/{\rm{Y}}\,{\rm{sole}}\times {\rm{T}}\,{\rm{main}}\,{\rm{crop}}/{\rm{Ti}}\\ {\rm{ATER}}\,{\rm{intercrop}} & = & {\rm{Y}}\,{\rm{intercrop}}/{\rm{Y}}\,{\rm{sole}}\times {\rm{T}}\,{\rm{intercrop}}/{\rm{Ti}}\end{array}$$

where T sole is the duration of growth cycle of main crop; T intercrop is the duration of growth cycle intercrop and Ti is the duration in days of the species with the longest growing period.

### Monetary Advantage Index (MAI)

For economic advantage of the intercropping system (MAI) was calculated as

$${\rm{MAI}}=\frac{({\rm{value}}\,{\rm{of}}\,{\rm{combined}}\,{\rm{intercrops}})\times ({\rm{LER}})}{{\rm{LER}}}$$

The higher the MAI value the more gainful is the cropping system^19^.

### Intercropping advantages (IA)

IA is the advantages or disadvantages of intercropping system depending on the sign of value, positive value mean advantages and vice versa. IA can be calculated by the following formula.

$${\rm{IA}}=[({{\rm{P}}}_{{\rm{a}}}/{{\rm{P}}}_{{\rm{a}}}+{{\rm{P}}}_{{\rm{b}}})\times {{\rm{AYL}}}_{{\rm{b}}}]+[({{\rm{P}}}_{{\rm{b}}}/{{\rm{P}}}_{{\rm{a}}}+{{\rm{P}}}_{{\rm{b}}})\times {{\rm{AYL}}}_{{\rm{a}}}]$$

In this equation, P_a_ is the price of species a, Pb is the price of species b, AYL_a_ is the partial actual yield loss or gain of species a and AYL_b_ is the partial actual yield loss or gain of species b.

### Land utilization efficiency (LUE)

By using ATER and LER values, the land utilization efficiency (LUE) was calculated according to equation as follows by

$${\rm{LUE}}=\frac{{\rm{LER}}\times {\rm{ATER}}}{2}\times 100$$

### System productivity index (SPI)

SPI is another pointer used to assess intercropping that standardizes the yield of the secondary crop in terms of the primary crop and is calculated as follow by^49^:

$${\rm{SPI}}=({{\rm{S}}}_{{\rm{m}}}/{{\rm{S}}}_{{\rm{i}}})\times {{\rm{Y}}}_{{\rm{m}}}+{{\rm{Y}}}_{{\rm{i}}}$$

where *S*_m_ and *S*_i_ are the average yields of main crop and intercrop under monoculture, respectively, and *Y*_m_ and *Y*_i_ are the average yields of main crop and intercrop under intercropping, respectively.

### Statistical analysis

Experiments were carried out for two years. However, year has no significant effect on competitive indices and yield of crop. Thus, the data of both years were combined for statistical analysis. Mean values were calculated for each of the competitive indices with respect to irrigation and intercropping system. Data were subjected to analysis of variance (ANOVA) according to the methods described in Steel and Torrie^50^ and treatment means were compared using the least significant difference (LSD) at P ≤ 0.05.

## Results

### Grains spike^−1^, 1000 grains weight (g) and grain yield of wheat (kg ha^−1^)

**Figure 1:**
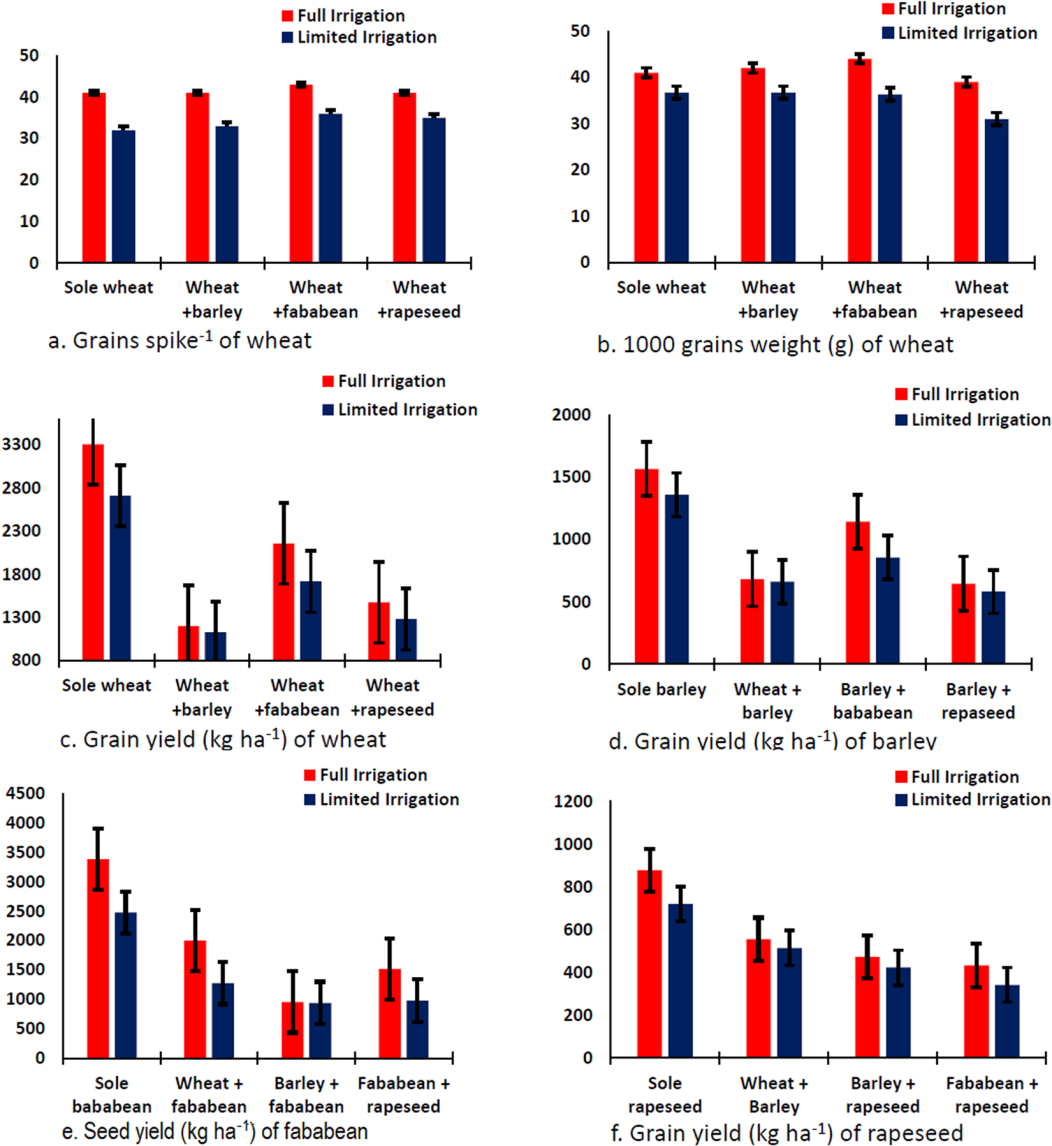
Interactive effect of irrigation and intercropping on winter crops (wheat, barley, fababean and rapeseed).

Data regarding grains spike^−1^, 1000 grains weight (TGW) and grain yield of wheat are presented in Table 3. Both irrigation and intercropping system and their interactive effect were significantly affected 1000 grains weight and grain yield of wheat. Maximum TGW and grain yield were recorded under full irrigated regime as compared with limited irrigation regime while number of grains spike^−1^ was not significantly affected by irrigation however under full irrigated regimes produced higher grains spike^−1^. In case of intercropping system higher grains spike^−1^ and TGW were recorded when wheat intercropped with fababean followed by wheat intercropped with rapeseed. Higher grain yield was recorded for wheat when grown as sole crop followed by wheat intercropped with fababean. Interactive effect of different irrigations regimes and intercropping system showed that wheat intercropped with fababean showed the most productive intercropping system in term of grains spike^−1^, TGW and grain yield (Fig. 1a–c) in wheat crop.

**Table 3:** Effect of different intercropping systems and irrigations on grains spike^−1^ or seeds/pod^−1^, 1000 grains/seeds weight (g) and grain/seed yield (kg ha^−1^) of wheat, barley, fababean and rapeseed, respectively.

Intercropping	Grains spike^−1^	1000 grains weight	Grain yield	Intercropping	Grains spike^−1^	1000 grains weight	Grain yield
Sole Wheat	36 c	38.8 b	3005 a	Sole Barley	33.3 ab	33.9 c	1460 a
Wheat + Barley	37 c	40.5ab	1163 d	Wheat + Barley	30.8 c	36.7 b	670 c
Wheat + Fababean	40 a	43.3 a	1963 b	Barley + Fababean	34.3 a	38.6 a	997 b
Wheat + Rapeseed	38 b	35.0 c	1377 c	Barley + Rapeseed	31.5 bc	36.3 b	613 d
Full irrigation	41 a	41.7 a	2033 a	Full irrigation	33.5	38.8 a	1007 a
Limited irrigated	35 b	35.2 b	1707 b	Limited irrigated	31.5	33.9 b	862 b
LSD _(0.05)_ for irrigation	1.7	0.8	99	LSD _(0.05)_ for irrigation	ns	0.9	61
LSD _(0.05)_ for intercropping	1.2	1.5	136	LSD _(0.05)_ for intercropping	1.8	1.3	63
LSD _(0.05)_ for interaction	1.7	2.1	190	LSD _(0.05)_ for interaction	ns	ns	88
**Intercropping**	**Grain pod**^**−1**^	**1000 seeds weight**	**Seed yield**	**Intercropping**	**Seeds pod**^**−1**^	**1000 seeds weight**	**Seed yield**
Sole Fababean	3.6 a	405	2927 a	Sole Rapeseed	136 a	4.6	782 a
Fababean + Wheat	3.6 a	390.7	1637 b	Rapeseed + Wheat	134 a	4.5	536 b
Fababean + Barley	3.6 a	393.7	945 d	Rapeseed + Barley	126 b	4.6	446 c
Fababean + Rapeseed	3.5 b	398.7	1246 c	Rapeseed + Fababean	123 b	4.4	386 c
Full irrigation	3.7	412.6 a	1962 a	Full irrigation	135 a	4.7 a	576 a
Limited irrigated	3.4	381.4 b	1416 b	Limited irrigated	125 b	4.3 b	499 b
LSD _(0.05)_ for intercropping	0.1	ns	67	LSD _(0.05)_ for intercropping	5.4	ns	22
LSD _(0.05)_ for irrigation	ns	16.3	86	LSD _(0.05)_ for irrigation	7.6	0.19	14
LSD _(0.05)_ for interaction	ns	ns	94	LSD _(0.05)_ for interaction	ns	ns	31

Note; ns stand for statistically non-significant at 5% probability.

### Grains spike^−1^, 1000 grains weight (g) and grain yield of barley (kg ha^−1^)

Data concerning grains spike^−1^, TGW and grain yield of barley are shown in Table 3. Data showed that intercropping was significantly affected grains spike^−1^, TGW and grain yield. TWG and grain yield were also significantly affected by different irrigation regimes while grains spike^−1^ was not significantly affected. Among different intercropping system higher grains spike^−1^ and TGW were recorded in case of wheat intercropped with fababean while minimum grains spike^−1^ were recorded when wheat intercropped with barley. Higher grain yield was recorded in case of sole barley followed by barely intercropped with wheat. Interactive effect of different intercropping system and irrigation regimes showed that highest grain yield was recorded when barley was grown as sole crop followed by barely intercropped with fababean under full irrigated regime (Fig. 1d).

### Seeds pod^−1^, 1000 seeds weight (g) and seed yield of fababean

Seeds pod^−1^ and seed yield were significantly affected by intercropping system while TGW was not statistically different (Table 3). Different irrigations regimes significantly affected grains pod^−1^, TGW and grain yield. Greater number of seeds pod^−1^, TGW and seed yield were recorded for full irrigated regime. Maximum seeds pod^−1^ were recorded for fababean intercropped with wheat which was statistically similar with intercropping of fababean with barley and sole fababean. Interactive effect of moisture regimes and intercropping for grain yield was found significant (Fig. 1e).

### Grains pod^−1^, 1000 grain weight (g) and grain yield of rapeseed

Data regarding pod plant^−1^ and grain yield of rapeseed was significantly affected by intercropping system and irrigation regimes (Table 3). Under full irrigation regime maximum grains pod^−1^, TWG and grain yield were produced as compared with limited irrigated regime. In case of intercropping system, maximum number of pods plant^−1^ were recorded for sole rapeseed crop followed by rapeseed intercropped with wheat and fababean, while minimum pods plant^−1^ were recorded when rapeseed intercropped with barley. Highest grain yield was recorded for sole rapeseed followed by rapeseed intercropped with wheat and fababean, respectively. Irrigation regimes and intercropping interaction had significantly affected grain yield of barley, higher grain yield was produced in intercropping system of rapeseed and fababean under full irrigated regime (Fig. 1f).

### Grains pod^−1^ and seeds head^−1^, 1000 grains/seeds weight (TGW/TSW) and grain yield of sorghum and pearl millet, and seed yield^−1^ of pigeonpea and mungbean

**Figure 2:**
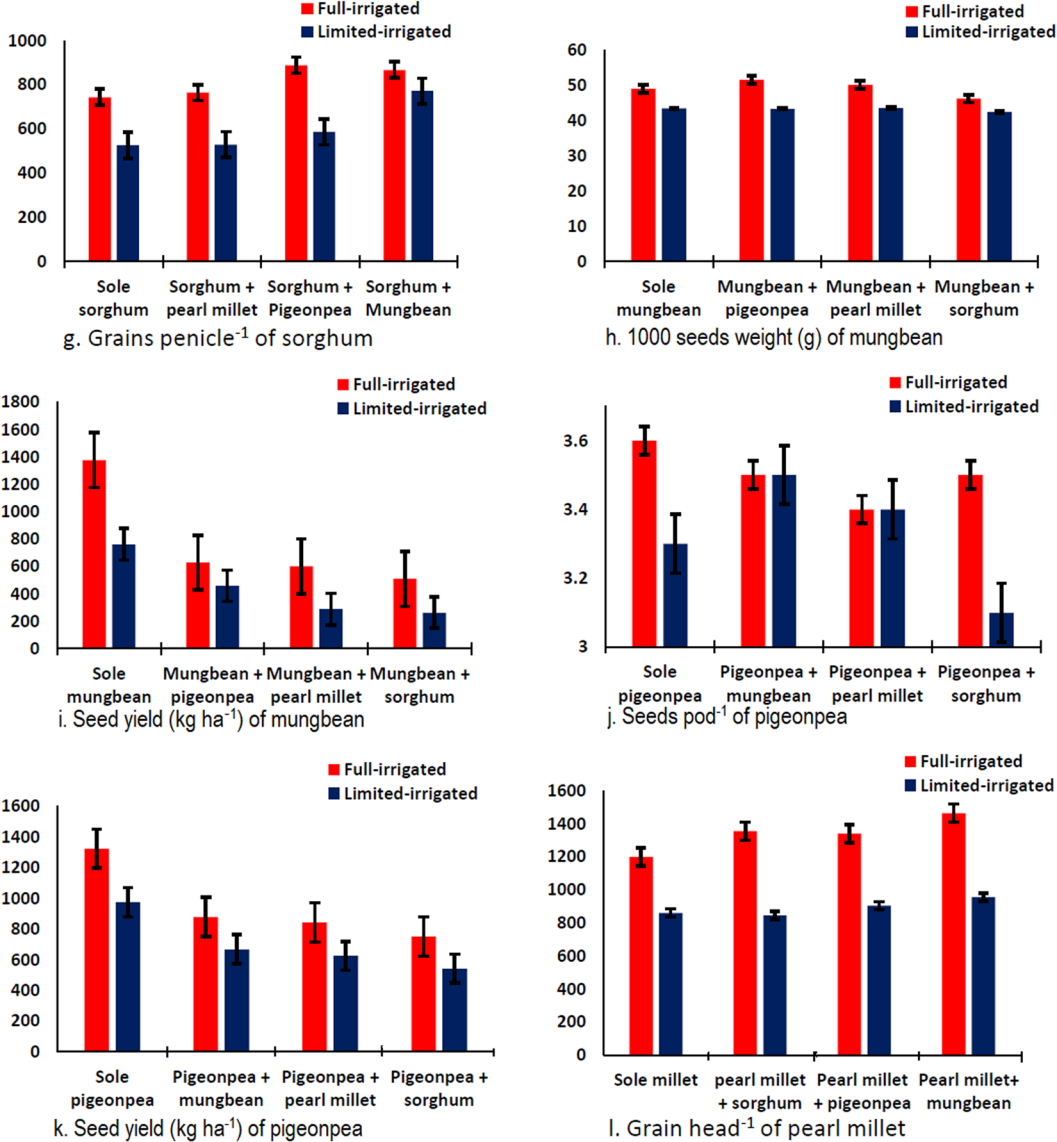
Interactive effect of irrigation and intercropping on summer crops (sorghum, mungbean, pigeonpea and pearl millets).

Data regarding grains head^−1^ or seeds pod^−1^, TGW and grain yield of pearl millet, sorghum, and seed yield of mungbean and pigeonpea are presented in Table 4. Data revealed that both, irrigation and intercropping were significantly affected grains head^−1^or seeds pod^−1^, TGW and grain/seed yield of all studied summer crops. All crops under full irrigated condition produced significantly higher grains head^−1^ or seeds pod^−1^, TGW and grain/seed yield than limited irrigated condition. All studied crops grown in intercropped produced comparatively higher grains head^−1^ or seeds pod^−1^ than sole crop except pigeonpea. Pearl millet intercropped with mungbean produced higher grains head^−1^ than intercropped with others crops or grown as sole crop. Sorghum intercropped with both legumes, produced higher grains head^−1^ than intercropped with pearl millet (Fig. 2g). Pigeonpea intercropped with mungbean produced higher grains pod^−1^ than intercropped with pearl millet or sorghum. Moreover sorghum/pearl millet intercropped with mungbean produced higher grains head^−1^ than sorghum/pearl millet intercropped with pigeonpea. Statistical analysis of the data of all crops revealed that irrigation and intercropping were significantly affected TGW of all crops except pearl millet, in which the effect of intercropping was found non-significant. Sorghum intercropped with mungbean produced higher TGW, followed by intercropping with pigeonpea which was statistically similar with intercropping with pearl millet or grown as sole crop (Table 4). Mungbean intercropped with pigeonpea produced higher TSW followed by intercropped with pearl millet or grown as sole crop while lower TSW was recorded in intercropping with sorghum (Fig. 2h). Mungbean intercropped with pigeonpea and/or pigeonpea intercropped with mungbean produced higher seed yield than intercropped with cereals (Fig. 2i). Pigeonpea intercropped with pearl millet produced higher seeds pod^−1^ (Fig. 2j) which was statistically similar with pigeonpea intercropped with mungbean and sorghum. On the other hand, pigeonpea intercropped with mungbean and millets produced higher seed yield (Fig. 2k). Additionally, mungbean/ pigeonpea intercropped with pearl millet produced higher seed yield as compared with mungbean/pigeonpea intercropped with sorghum (Fig. 2l).

**Table 4:** Effect of different intercropping systems and irrigations on grains/seeds head^−1^/pod^−1^, 1000 grains/seeds weight (g) and grain/seed yield (kg ha^−1^) of pearl millet, sorghum, pigeonpea and mungbean, respectively.

Intercropping	Grains head^−1^	1000 grains weight (g)	Grain yield	Intercropping	Grains head^−1^	1000 grains weight (g)	Grain yield
Sole Millet	1029 c	10.8	1175 a	Sole Sorghum	635 d	19.8 b	1423 a
Millet + Sorghum	1100 b	10.8	594 d	Sorghum + Millet	647 c	19.6 b	809 c
Millet + Pigeonpea	1121 b	10.9	698 c	Sorghum + Pigeonpea	738 b	19.9 b	848 c
Millet + Mungbean	1210 a	11.2	776 b	Sorghum + Mungbean	820 a	22.1 a	935 b
Full irrigation	1339 a	11.6 a	991 a	Full irrigation	816 a	20.8 a	1164 a
Limited irrigated	891 b	10.2 b	630 b	Limited irrigated	604 b	19.9 b	844 b
LSD _(0.05)_ for irrigation	67	0.29	46	LSD _(0.05)_ for irrigation	15.1	0.4	70
LSD _(0.05)_ for intercropping	40	ns	58	LSD _(0.05)_ for intercropping	18.3	0.6	55
LSD _(0.05)_ for interaction	56	ns	ns	LSD _(0.05)_ for interaction	25.8	ns	ns
**Intercropping**	**Seeds pod**^**−1**^	**1000 seeds weight**	**Seed yield**	**Intercropping**	**Seeds pod**^**−1**^	**1000 Seeds weight**	**Seed yield**
Sole Pigeonpea	3.5 a	57.5 b	1149 a	Sole Mungbean	7.4 b	46.3 b	1069 a
Pigeonpea + Mungbean	3.5 b	59.1 a	773 b	Mungbean + Pigeonpea	8.1 a	47.5 a	544 b
Pigeonpea + Pearl millet	3.4 c	60.0 a	735 c	Mungbean + Pearl millet	7.6 ab	46.9 b	445 c
Pigeonpea + Sorghum	3.3 d	59.6 a	647 d	Mungbean + Sorghum	7.2 b	44.4 c	387 d
Full irrigation	3.5 a	64.7 a	950 a	Full irrigation	8.3 a	49.3 a	780 a
Limited irrigation	3.3 b	53.4 b	703 b	Limited irrigated	6.8 b	43.2 b	443 b
LSD _(0.05)_ for intercropping	0.10	1.0	37	LSD _(0.05)_ for intercropping	0.6	1.0	31
LSD _(0.05)_ for irrigation	0.11	0.9	41	LSD _(0.05)_ for irrigation	0.3	0.6	36
LSD _(0.05)_ for interaction	0.15	1.5	52	LSD _(0.05)_ for interaction	ns	1.5	44

Note; ns stand for statistically non-significant at 5% probability.

### Land equivalent ratio (LER)

LER is intercropping terminology using to assess the land utilization under intercropping system than monocropping system. Among winter crops under full irrigated condition only intercropping of wheat with fababean showed higher LER than one with the remain all showed less than one which mean that only intercropping of wheat with fababean have intercropping advantages under full irrigated condition (Table 5). In case of limited irrigated condition all intercropping system had higher or equal LER to sole cropping which than under limited irrigated condition all the studied combination of crops had intercropping advantages. On the other side intercropping of summer crops showed higher LER than one except intercropping of mungbean with sorghum and pearl millet under both water regimes. The partial value of LER showed that sorghum had taken highest benefit of intercropping as when intercropped with mungbean. Partial LER greater than 0.5 showing intercropping advantages over monocropping. Table 5 showed that intercropping of summer cereals with legumes crops taking highest benefits of intercropping. In summer, cereal legumes intercropping system, intercropping pigeonpea with pearl millet and sorghum were the most promising system under both irrigated and limited irrigated condition by the best land utilization over monocropping.

**Table 5:** Effect of irrigation regimes on land equivalent ratio in different intercropping system.

Land equivalent ratio										
Winter Season						Summer Season				
Irrigation	Main crop	Intercrop	Main crop	Intercrop	System	Main crop	Intercrop	Main crop	Intercrop	System
Full	Wheat	Barley	0.42 d	0.43 c	0.81 c	Sorghum	Pigeonpea	0.62 b	0.57 b	1.19 a
Full	Wheat	Fababean	0.53 c	0.62 ab	1.12 ab	Sorghum	Pearl millet	0.58 bc	0.54 b	1.12 ab
Full	Wheat	Rapeseed	0.41 d	0.63 a	1.03 b	Sorghum	Mungbean	0.68 a	0.37 d	1.05 b
Full	Barley	Fababean	0.53 c	0.33 c	0.84 c	Pearl millet	Pigeonpea	0.63 b	0.64 a	1.26 a
Full	Barley	Rapeseed	0.41 d	0.53 b	0.92 bc	Pearl millet	Mungbean	0.70 a	0.43 cd	1.13 ab
Full	Fababean	Rapeseed	0.43 d	0.51 bc	0.91 bc	Pigeonpea	Mungbean	0.66 ab	0.45 c	1.11 ab
Limited	Wheat	Barley	0.52 c	0.62 ab	1.13 ab	Sorghum	Pigeonpea	0.56 c	0.56 b	1.12 ab
Limited	Wheat	Fababean	0.72 a	0.63 a	1.31 a	Sorghum	Pearl millet	0.55 c	0.46 c	1.01 b
Limited	Wheat	Rapeseed	0.61 b	0.63 a	1.22 ab	Sorghum	Mungbean	0.62 b	0.35 cd	0.97 b
Limited	Barley	Fababean	0.63 b	0.42 c	1.13 ab	Pearl millet	Pigeonpea	0.55 c	0.64 a	1.19 a
Limited	Barley	Rapeseed	0.63 b	0.63 a	1.21 ab	Pearl millet	Mungbean	0.61 cd	0.38 cd	0.99 b
Limited	Fababean	Rapeseed	0.52 c	0.62 ab	1.02 b	Pigeonpea	Mungbean	0.68 ab	0.60 ab	1.28 a
**Intercropping means**										
	Wheat	Barley	0.47 d	0.52 e	0.97 b	Sorghum	Pigeonpea	0.59 b	0.56 b	1.15 a
	Wheat	Fababean	0.62 a	0.62 b	1.21 a	Sorghum	Pearl millet	0.56 c	0.50 d	1.07 bc
	Wheat	Rapeseed	0.51 c	0.63 a	1.13 ab	Sorghum	Mungbean	0.65 a	0.36 f	1.01 b
	Barley	Fababean	0.58 b	0.37 f	0.98 b	Pearl millet	Pigeonpea	0.59 b	0.64 a	1.22 a
	Barley	Rapeseed	0.52 c	0.58 c	1.07 b	Pearl millet	Mungbean	0.66 a	0.41 e	0.81 c
	Fababean	Rapeseed	0.48 d	0.57 d	0.97b	Pigeonpea	Mungbean	0.67 a	0.53 c	1.20 a
**Irrigation means**										
Full irrigation			0.45 b	0.51 b	0.94 b	Full irrigation		0.64 a	0.50	1.14 a
Limited irrigation			0.60 a	0.59 a	1.17 a	Limited irrigation		0.59 b	0.50	1.01 b
LSD _(0.05)_ for intercropping			0.02	0.05	0.11	LSD _(0.05)_ for intercropping		0.04	0.03	0.16
LSD _(0.05)_ for Irrigation			0.12	0.04	0.10	LSD _(0.05)_ for Irrigation		0.11	ns	0.08
LSD _(0.05)_ for irrigation × intercropping			0.03	0.10	0.21	LSD _(0.05)_ for irrigation × intercropping		0.06	0.07	0.21

Note; ns stand for statistically non-significant at 5% probability

### Aggressivity

Aggressivity is a competition index used to describe the relative yield increase in crop “a” than crop “b” in an intercropping system. Aggressivity of the winter season showed that all crops combination are not similar in competition under both moisture conditions, higher aggressivity was recorded for barley grown in intercropping with fababean under both water regimes followed by wheat intercropped with rapeseed then wheat intercropped with fababean under high and low moist condition, respectively, while lowest aggressivity was recorded under high moist condition (Table 6). In case of wheat intercropped with barley and fababean, while in case of limited Irrigated condition lowest aggressivity was determined in intercropping of barley with wheat and rapeseed. Aggressivity value of the summer intercropping also revealed that companion crop did not compete equally. Sorghum or pearl millet intercrop with mungbean shown highest positive aggressivity over mungbean. Sorghum/ pearl millet intercropped with pigeonpea showed least positive aggressivity value which mean that these crops are compatible with each other. Under low moisture condition pigeonpea intercrop with mung bean showed least aggressivity. Among winter cereals barley is the strongest competitor with other crops no one is able to compress it expect rapeseed which slight aggressiveness over barley under both water regimes.

**Table 6:** Effect of irrigation regimes on aggressivity in different intercropping systems.

Aggressivity								
Winter Season					Summer Season			
Irrigation	Main crop	Intercrop	Main crop	Intercrop	Main crop	Intercrop	Main crop	Intercrop
Full	Wheat	Barley	−0.07 e	0.07 d	Sorghum	Pigeonpea	0.04 f	−0.04 b
Full	Wheat	Fababean	−0.07 e	0.07 d	Sorghum	Pearl millet	0.09 e	−0.07 c
Full	Wheat	Rapeseed	−0.15 h	0.15 a	Sorghum	Mungbean	0.23 b	−0.23 f
Full	Barley	Fababean	0.22 a	−0.22 h	Pearl millet	Pigeonpea	0.03 f	−0.03 b
Full	Barley	Rapeseed	−0.13 g	0.12 b	Pearl millet	Mungbean	0.18 c	−0.18 e
Full	Fababean	Rapeseed	−0.10 f	0.09 c	Pigeonpea	Mungbean	0.13 d	−0.13 d
Limited	Wheat	Barley	−0.01 d	0.01 e	Sorghum	Pigeonpea	0.03 f	−0.03 b
Limited	Wheat	Fababean	0.14 b	−0.14 g	Sorghum	Pearl millet	0.15 cd	−0.15 d
Limited	Wheat	Rapeseed	0.07 c	−0.07 f	Sorghum	Mungbean	0.27 a	−0.27 g
Limited	Barley	Fababean	0.21 a	−0.21 h	Pearl millet	Pigeonpea	0.09 e	−0.09 c
Limited	Barley	Rapeseed	−0.02 d	0.02 e	Pearl millet	Mungbean	0.09 e	−0.09 c
Limited	Fababean	Rapeseed	−0.10 f	0.10 bc	Pigeonpea	Mungbean	0.00 i	0.00 a
**Intercropping means**								
	Wheat	Barley	−0.04 c	0.04 b	Sorghum	Pigeonpea	0.04 e	−0.03 a
	Wheat	Fababean	0.04 b	−0.04 c	Sorghum	Pearl millet	0.11 c	−0.11 c
	Wheat	Rapeseed	−0.04 c	0.04 b	Sorghum	Mungbean	0.25 a	−0.25 e
	Barley	Fababean	0.22 a	−0.21 d	Pearl millet	Pigeonpea	0.06	−0.06 b
	Barley	Rapeseed	−0.08 d	0.07 a	Pearl millet	Mungbean	0.13 b	−0.14 d
	Fababean	Rapeseed	−0.10 e	0.09 a	Pigeonpea	Mungbean	0.07 d	−0.07 b
**Irrigation means**								
Full irrigation			−0.05 b	0.05 a	Full irrigation		0.11	−0.12 b
Limited irrigation			0.05 a	−0.05 b	Limited irrigation		0.11	−0.11 a
LSD _(0.05)_ for intercropping			0.01	0.01	LSD _(0.05)_ for intercropping		0.03	0.02
LSD _(0.05)_ for Irrigation			0.01	0.01	LSD _(0.05)_ for Irrigation		ns	0.01
LSD _(0.05)_ for irrigation × intercropping			0.02	0.03	LSD _(0.05)_ for irrigation × intercropping		0.04	0.03

Note; ns stand for statistically non-significant at 5% probability.

### Competition ratio (CR)

Competition ratio showed different response of winter crops under both irrigation regimes. Intercropped with legumes crops, rapeseed was the dominant crop than fababean, having highest CR over winter cereals i.e. wheat and barley, while in case of winter cereals, barley was the most dominant species over wheat, having higher CR than wheat, under intercropping system of wheat/barley with fababean and rapeseed (Table 7). In case of wheat- fababean/ rapeseed intercropping system, rapeseed and fababean were the dominant species over wheat under full irrigated condition, it might be due the suitable moisture, quick initial growth of the legumes and friendlily condition with wheat crop as compared with barley, which have some allelopathic effect on complain crops. In case of summers cereal, sorghum was the most dominant crop than pearl millet over legumes crops specially mung bean having highest CR under both water regimes. In case of summer legumes pigeonpea was the most competitive crop than mung bean with pearl millet and sorghum, respectively.

**Table 7:** Effect of irrigation regimes on competition ratio in different intercropping systems.

Competition ratio								
Winter Season					Summer Season			
Irrigation	Main crop	Intercrop	Main crop	Intercrop	Main crop	Intercrop	Main crop	Intercrop
Full	Wheat	Barley	0.83 de	1.20 ab	Sorghum	Pigeonpea	1.06 bc	0.94 ab
Full	Wheat	Fababean	0.89 de	1.13 b	Sorghum	Pearl millet	1.15 bc	0.87 ab
Full	Wheat	Rapeseed	0.75 e	1.34 a	Sorghum	Mungbean	1.52 a	0.66 b
Full	Barley	Fababean	1.79 a	0.56 e	Pearl millet	Pigeonpea	1.05 bc	0.95 ab
Full	Barley	Rapeseed	0.78 e	1.28 ab	Pearl millet	Mungbean	1.37 ab	0.66 b
Full	Fababean	Rapeseed	0.83	1.23 ab	Pigeonpea	Mungbean	1.23 b	0.81 b
Limited	Wheat	Barley	0.99 dc	1.01 bc	Sorghum	Pigeonpea	1.07 bc	0.94 ab
Limited	Wheat	Fababean	1.25 c	0.80 c	Sorghum	Pearl millet	1.16 bc	0.86 ab
Limited	Wheat	Rapeseed	1.12 c	0.89 c	Sorghum	Mungbean	1.30 b	0.77 b
Limited	Barley	Fababean	1.49 b	0.67 d	Pearl millet	Pigeonpea	1.09 bc	0.92 ab
Limited	Barley	Rapeseed	0.96 d	1.04 bc	Pearl millet	Mungbean	1.18 bc	0.85 ab
Limited	Fababean	Rapeseed	0.83 de	1.21 ab	Pigeonpea	Mungbean	1.01 c	0.99 a
**Intercropping means**								
	Wheat	Barley	0.91 c	1.10 a	Sorghum	Pigeonpea	1.06 c	0.94 a
	Wheat	Fababean	1.07 b	0.97 b	Sorghum	Pearl millet	1.16 c	0.87 a
	Wheat	Rapeseed	0.93 c	1.11 a	Sorghum	Mungbean	1.41 a	0.71 b
	Barley	Fababean	1.64 a	0.61 c	Pearl millet	Pigeonpea	1.07 c	0.93 a
	Barley	Rapeseed	0.87 c	1.16 a	Pearl millet	Mungbean	1.27 b	0.75 b
	Fababean	Rapeseed	0.83 c	1.22 a	Pigeonpea	Mungbean	1.12 c	0.90 a
**Irrigation means**								
Full irrigation			0.98 b	1.12 a	Full irrigation		1.23 a	0.81 b
Limited irrigation			1.11 a	0.94 b	Limited irrigation		1.13 b	0.89 a
LSD _(0.05)_ for intercropping			0.11	0.13	LSD _(0.05)_ for intercropping		0.11	0.11
LSD _(0.05)_ for irrigation			0.11	0.13	LSD _(0.05)_ for irrigation		0.08	0.05
LSD _(0.05)_ for irrigation × intercropping			0.14	0.18	LSD _(0.05)_ for irrigation × intercropping		0.19	0.15

### Relative crowding co-efficient (K)

Relative Crowding Co-Efficient (K) is an intercropping index which evaluating and comparing the competitive ability of one species to the other in a mixture. To calculate the relative dominance of crop species over the other spices of crop in intercropping system, relative crowding coefficient is the best option (Table 8). Under irrigated condition of winter crops K showed that intercropping of wheat with fababean and rapeseed were the most successfully combination among others. The lowest value for K was recorded for intercropping system of barley with wheat and fababean under irrigated condition, while under limited irrigated condition highest K valued was calculated for intercropping of wheat with fababean, and barley with rapeseed. While on the other hand, intercropping of pigeonpea with mungbean, pearl millet and sorghum showed highest intercropping system due highest value for K (pigeonpea + mungbean > pigeonpea + pearl millet > pigeonpea + sorghum) under irrigated condition, while under limited irrigated condition highest intercropping of pigeonpea + mungbean showed higher value for K followed by pearl millet intercropped with mungbean while intercropping of sorghum with pigeonpea, pearl millet, mungbean showed no considerable increase than one, which mean intercropping of these crops under limited irrigated condition similar to the their respective monocropping.

**Table 8:** Effect of irrigation regimes on relativecrowding  co-efficient (K) in different intercropping systems.

Relative **Crowding** crowding Co-Efficient (K)										
Winter Season						Summer Season				
Irrigation	Main crop	Intercrop	Main crop	Intercrop	System	Main crop	Intercrop	Main crop	Intercrop	System
Full	Wheat	Barley	0.61 i	0.82 d	0.50 g	Sorghum	Pigeonpea	1.71 b	1.51 a	2.58 b
Full	Wheat	Fababean	1.11 e	1.43 ab	1.59 bc	Sorghum	Pearl millet	1.32 c	0.91 c	1.21 e
Full	Wheat	Rapeseed	0.82 f	1.53 a	1.26 d	Sorghum	Mungbean	2.12 a	0.82 c	1.73 d
Full	Barley	Fababean	1.02 e	0.42 e	0.42 g	Pearl millet	Pigeonpea	1.63 b	1.52 a	2.47 b
Full	Barley	Rapeseed	0.72 fi	1.12 c	0.81 ef	Pearl millet	Mungbean	1.91 ab	0.81 c	1.54 d
Full	Fababean	Rapeseed	0.71 fi	1.03 c	0.73 f	Pigeonpea	Mungbean	2.22 a	1.32 b	2.94 a
Limited	Wheat	Barley	1.22 de	1.31 b	1.60 bc	Sorghum	Pigeonpea	1.11 c	0.91 c	1.00 e
Limited	Wheat	Fababean	2.52 a	1.32 b	3.33 a	Sorghum	Pearl millet	1.22 c	0.91 c	1.12 e
Limited	Wheat	Rapeseed	1.62 c	1.23 bc	1.99 b	Sorghum	Mungbean	1.31 c	0.81 c	1.06 e
Limited	Barley	Fababean	1.91 b	0.81 d	1.56 c	Pearl millet	Pigeonpea	1.11 c	0.91 c	1.01 e
Limited	Barley	Rapeseed	1.41 d	1.51 a	2.13 bc	Pearl millet	Mungbean	1.31 c	0.91 c	1.19 e
Limited	Fababean	Rapeseed	0.82 f	1.23 b	0.99 e	Pigeonpea	Mungbean	1.51 bc	1.51 a	2.28 c
**Intercropping means**										
	Wheat	Barley	0.91e	1.06 c	1.04 c	Sorghum	Pigeonpea	1.41 c	1.21 b	1.79 b
	Wheat	Fababean	1.82 a	1.38 a	2.46 a	Sorghum	Pearl millet	1.27 c	0.91 c	1.16 d
	Wheat	Rapeseed	1.22 c	1.38 a	1.62 b	Sorghum	Mungbean	1.71 b	0.81 d	1.39 c
	Barley	Fababean	1.46 b	0.61 d	0.99 d	Pearl millet	Pigeonpea	1.37 c	1.21 b	1.74 b
	Barley	Rapeseed	1.06 d	1.31 a	1.47 c	Pearl millet	Mungbean	1.61 b	0.86 c	1.37 c
	Fababean	Rapeseed	0.77 f	1.13 bc	0.86 d	Pigeonpea	Mungbean	1.87 a	1.42 a	2.61 a
**Irrigation means**										
Full irrigation Limited irrigation			0.83 b	1.06 b	0.88 b	Full irrigation		1.82 a	1.15 a	2.08 a
			1.58 a	1.23 a	1.93 a	Limited irrigation		1.26 b	0.99 b	1.28 b
LSD _(0.05)_ for intercropping			0.14	0.13	0.15	LSD _(0.05)_ for intercropping		0.15	0.14	0.20
LSD _(0.05)_ for irrigation			0.32	0.13	0.45	LSD _(0.05)_ for irrigation		0.43	0.10	0.45
LSD _(0.05)_ for irrigation × intercropping			0.20	0.19	0.22	LSD _(0.05)_ for irrigation × intercropping		0.23	0.18	0.25

### Actual yield loss (AYL)

The AYL is the proportionate yield loss or gain of intercrops compared to sole crop. AYL give more accurate evidence about intercropping than the other indexes on the intra- and inter-specific competition and behavior of the component crops. In case of winter crops, intercropping of barley with wheat/ fababean/ rapeseed showed a disadvantage of intercropping system due to negative value for ALY under full irrigated condition, while under limited irrigated all the intercropping combination have positive value for intercropping (Table 9). Highest ALY was observed for wheat and fababean when intercropped with barley, under both water regimes intercropping of wheat with fababean was the most successfully intercropping system in tram of positive AYL value, followed by wheat intercropped with rapeseed. In case of summer crops, all intercropping system have positive ALY value except intercropping of sorghum with mungbean under limited irrigated condition. In tram of partial ALS value highest benefit had taken by sorghum and pearl millet when intercropped with mungbean, while considerably suppressed the growth of mungbean. Pigeonpea was the strongest competitor crop in these intercropping system by maintaining positive value of AYL as intercropped with strongest summer cereals i.e., sorghum and pearl millet under both water regimes.

**Table 9:** Effect of irrigation regimes on actual yield loss in different intercropping systems.

Actual yield loss										
Winter Season						Summer Season				
Irrigation	Main crop	Intercrop	Main crop	Intercrop	System	Main crop	Intercrop	Main crop	Intercrop	System
Full	wheat	barley	−0.31 d	−0.13 c	−0.43 e	Sorghum	pigeonpea	0.21b	0.21 b	0.42 b
Full	wheat	fababean	0.02 c	0.21 ab	0.23 c	Sorghum	pearl millet	0.21 b	0.11 b	0.22 c
Full	wheat	rapeseed	−0.11 cd	0.23 a	0.14 dc	Sorghum	mungbean	0.44 a	−0.31 e	0.13 cd
Full	barley	fababean	0.02 c	−0.42 d	−0.44 e	Pearl millet	pigeonpea	0.21 b	0.32 a	0.53 ab
Full	barley	rapeseed	−0.22 d	0.11 ab	−0.13 d	Pearl millet	mungbean	0.41 a	−0.31 d	0.12 cd
Full	fababean	rapeseed	−0.21 d	0.03 c	−0.14 d	Pigeonpea	mungbean	0.31 ab	−0.11 c	0.20 cd
Limited	wheat	barley	0.11 bc	0.13 b	0.24 c	Sorghum	pigeonpea	0.12 b	0.11 b	0.22 c
Limited	wheat	fababean	0.42 a	0.12 ab	0.64 a	Sorghum	pearl millet	0.12 b	−0.10 c	0.01 d
Limited	wheat	rapeseed	0.22 b	0.12 ab	0.44 b	Sorghum	mungbean	0.21 b	−0.32 e	−0.11 d
Limited	barley	fababean	0.31 ab	−0.12	0.23 c	Pearl millet	pigeonpea	0.11 b	0.31 ab	0.42 b
Limited	barley	rapeseed	0.22 b	0.21 ab	0.42 bc	Pearl millet	mungbean	0.22 b	−0.20 d	0.03 d
Limited	fababean	rapeseed	−0.12 cd	0.11 ab	0.03 d	Pigeonpea	mungbean	0.41 a	0.21 b	0.62 a
**Intercropping means**										
	wheat	barley	−0.10 c	0.00 b	−0.10 d	Sorghum	pigeonpea	0.16 b	0.16 b	0.32 b
	wheat	fababean	0.22 a	0.17 a	0.44 a	Sorghum	pearl millet	0.17 b	0.01 c	0.12 c
	wheat	rapeseed	0.06 b	0.17 a	0.29 b	Sorghum	mungbean	0.32 a	−0.31d	0.01
	barley	fababean	0.17 a	−0.27 c	−0.10 d	Pearl millet	pigeonpea	0.16 b	0.32 a	0.48 a
	barley	rapeseed	0.00 bc	0.16 a	0.14 c	Pearl millet	mungbean	0.31 a	−0.25 d	0.07
	fababean	rapeseed	−0.16 c	0.07 ab	−0.05 d	Pigeonpea	mungbean	0.36 a	0.05 c	0.41 a
**Irrigation means**										
Full irrigation			−0.13 b	0.00 a	−0.13 b		Full irrigation	0.30 a	−0.01	0.27 a
Limited irrigation			0.19 a	0.09 b	0.33 a		Limited irrigation	0.20 b	0.00	0.20 b
LSD _(0.05)_ for intercropping			0.11	0.10	0.15		LSD _(0.05)_ for intercropping	0.10	0.09	0.15
LSD _(0.05)_ for irrigation			0.19	0.01	0.12		LSD _(0.05)_ for irrigation	0.09	ns	0.04
LSD _(0.05)_ for irrigation × intercropping			0.18	0.15	0.20		LSD _(0.05)_ for irrigation × intercropping	0.16	0.11	0.19

Note; ns stand for statistically non-significant at 5% probability

### Area time equivalent ratio (ATER)

Data regarding ATER of the both winter and summer crops showed in Table 10. ATER provides more realistic comparison of the yield advantage of intercropping over sole cropping in terms of variation. in time taken by the component crops of different intercropping systems. In all the treatments, the ATER values were lesser than LER values indicating the over estimation of resource utilization. ATER is free from problems of over estimation of resource utilization contrary to LEA. In case of winter crops all intercropping combination have less than one ATER except wheat intercropped fababean and rapeseed under both water regimes and wheat intercropped with barley intercropped with rapeseed and wheat under limited irrigation condition only (Table 10). While in case of summer season crops, ATER of all intercropping system showed less than one except sorghum intercropped with pearl millet under full irrigated condition and pigeonpea intercropped with mung bean under limited irrigated condition. All the intercropping system which have less than one ATER value had a disadvantage of intercropping in term of field occupation time.

**Table 10:** Effect of irrigation regimes on area time equivalent ratio in different intercropping systems.

Area time equivalent ratio						
Winter Season				Summer Season		
**Irrigation**	Main crop	Intercrop	System	**Main crop**	**Intercrop**	**System**
Full	wheat	barley	0.78 d	Sorghum	pigeonpea	0.99 b
Full	wheat	fababean	1.09 bc	Sorghum	pearl millet	1.15 a
Full	wheat	rapeseed	1.01 c	Sorghum	mungbean	0.99 b
Full	barley	fababean	0.67 e	Pearl millet	pigeonpea	0.95 bc
Full	barley	rapeseed	0.83 d	Pearl millet	mungbean	0.77
Full	fababean	rapeseed	0.82 d	Pigeonpea	mungbean	0.90 bc
Limited	wheat	barley	1.07 bc	Sorghum	pigeonpea	0.91 bc
Limited	wheat	fababean	1.28 a	Sorghum	pearl millet	1.10 b
Limited	wheat	rapeseed	1.15 bc	Sorghum	mungbean	0.86 c
Limited	barley	fababean	0.93 cd	Pearl millet	pigeonpea	0.92 bc
Limited	barley	rapeseed	1.05 bc	Pearl millet	mungbean	0.91 bc
Limited	fababean	rapeseed	0.88 d	Pigeonpea	mungbean	1.01 b
**Intercropping means**						
	wheat	barley	0.92 c	Sorghum	pigeonpea	0.95 b
	wheat	fababean	1.19 a	Sorghum	pearl millet	1.13 a
	wheat	rapeseed	1.08 b	Sorghum	mungbean	0.92 b
	barley	fababean	0.80 d	Pearl millet	pigeonpea	0.94 b
	barley	rapeseed	0.94 c	Pearl millet	mungbean	0.84 c
	fababean	rapeseed	0.85 d	Pigeonpea	mungbean	0.95 b
**Irrigation means**						
Full irrigation			0.86 b	Full irrigation		0.96 a
Limited irrigation			1.06 a	Limited irrigation		0.95 b
LSD _(0.05)_ for intercropping			0.07	LSD _(0.05)_ for intercropping		0.08
LSD _(0.05)_ for irrigation			0.13	LSD _(0.05)_ for irrigation		0.01
LSD _(0.05)_ for irrigation × intercropping			0.11	LSD _(0.05)_ for irrigation × intercropping		0.13

### Land utilization efficiency (LUE)

An intercropping system utilization of land is the main indicator which show the efficiency of an intercropping system. LUR value greater than 50 showed advantages of intercropping over mono cropping. Highest LUE value was recoded for wheat intercropped with fababean followed by wheat intercropped with rapeseed under both water regimes. Barley intercropped with wheat, fababean and rapeseed showed lowest land utilization efficiency under full irrigated condition, while under limited irrigated condition a little increase was observed (Table 11). On the other hand, summer crops all most all crops showed higher LUE than 50 except sorghum/millet intercropped with mungbean, with highest LUE value for intercropping of pigeonpea intercropped with mungbean under limited irrigated condition and sorghum intercropped with pearl millet under full irrigated condition followed by pearl millet intercropped with pigeonpea.

**Table 11:** Effect of irrigation regimes on land utilization efficiency in different intercropping systems.

Land utilization efficiency						
Winter Season				Summer Season		
Irrigation	Main crop	Intercrop	System	Main crop	Intercrop	System
Full	wheat	barley	31 b	Sorghum	pigeonpea	59 ab
Full	wheat	fababean	60 ab	Sorghum	pearl millet	64 a
Full	wheat	rapeseed	50 b	Sorghum	mung bean	51 ab
Full	barley	fababean	27 b	Pearl millet	pigeonpea	60 a
Full	barley	rapeseed	36 b	Pearl millet	mung bean	43 b
Full	fababean	rapeseed	37 b	Pigeonpea	mung bean	50 b
Limited	wheat	barley	59 ab	Sorghum	pigeonpea	51 ab
Limited	wheat	fababean	83 a	Sorghum	pearl millet	55 ab
Limited	wheat	rapeseed	69 ab	Sorghum	mung bean	42 b
Limited	barley	fababean	51 b	Pearl millet	pigeonpea	55 ab
Limited	barley	rapeseed	62 ab	Pearl millet	mung bean	45 b
Limited	fababean	rapeseed	44 b	Pigeonpea	mung bean	65 a
**Intercropping means**						
	wheat	barley	45 b	Sorghum	pigeonpea	55 ab
	wheat	fababean	72 a	Sorghum	pearl millet	60 a
	wheat	rapeseed	60 ab	Sorghum	mung bean	47 b
	barley	fababean	39 b	Pearl millet	pigeonpea	58 a
	barley	rapeseed	49 b	Pearl millet	mung bean	44 b
	fababean	rapeseed	40 b	Pigeonpea	mung bean	57 a
**Irrigation means**						
Full irrigation			40 b	Full irrigation		55 a
Limited irrigation			61 a	Limited irrigation		52 b
LSD _(0.05)_ for intercropping			18	LSD _(0.05)_ for intercropping		9
LSD _(0.05)_ for irrigation			17	LSD _(0.05)_ for irrigation		2
LSD _(0.05)_ for irrigation × intercropping			31	LSD _(0.05)_ for irrigation × intercropping		15

### Intercropping advantages (IA)

Intercropping advantage (IA) is also an indicator of the economic feasibility of intercropping systems. IA of the data showed that intercropping system of wheat with fababean and rapeseed had highest intercropping advantages over monoculture as showed by their positive for IA under both water regimes, followed by wheat intercropped with rapeseed while the rest of intercropping system had a disadvantages of intercropping under full irrigated condition, while under limited irrigated condition all of the intercropping system had intercropping advantages except fababean intercropped with barley and rapeseed (Table 12). Barley intercropped with wheat show negative IA for both component crops under full irrigated condition which mean wheat and barley intercropping system under normal water condition had disadvantages of intercropping. In case of wheat intercropped with rapeseed highest advantages of intercropping system had taken by rapeseed due to it high positive IA value while wheat had a disadvantages of intercropping but the overall system had positive value foe IA due high price of rapeseed which overcome the loss of wheat during intercropping system. Intercropping systems of summer crops showed positive value for IA except mungbean intercropped with sorghum and pearl millet under both water regimes. Sorghum and pearl millet intercropped with mungbean got highest beneficent of intercropping system by getting highest positive value for IA while the growth of mung bean was highly suppressed, which conform by the highest negative value of IA, but the overall system of had a positive value which mean increase in sorghum and pearl millet yield as result of intercropping with mungbean compensated mungbean yield reduction. While intercropping of pigeonpea with sorghum or pearl millet showed strongest competitive ability due to it comparatively high stature as compared with mung bean and branched nature.

**Table 12:** Effect of irrigation regimes on intercropping advantages in different intercropping systems.

Intercropping advantages										
Winter Season						Summer Season				
Irrigation	Main crop	Intercrop	Main crop	Intercrop	System	Main crop	Intercrop	Main crop	Intercrop	System
Full	wheat	barley	−420 i	−160 j	−580 f	Sorghum	pigeonpea	450 c	250 c	700 c
Full	wheat	fababean	0.5 e	1000 a	1000 a	Sorghum	pearl millet	450 c	200 c	650 d
Full	wheat	rapeseed	−140 g	640 b	500 b	Sorghum	mung bean	900 ab	−675 f	225 e
Full	barley	fababean	0.002 f	−2000 k	−2000 h	Pearl millet	pigeonpea	400 c	750 a	1150 b
Full	barley	rapeseed	−321 h	319 e	−1 d	Pearl millet	mung bean	799 b	−676 f	124 e
Full	fababean	rapeseed	−1001 j	−1 i	−1001 g	Pigeonpea	mung bean	750 b	−226 d	525 d
Limited	wheat	barley	141 d	161 f	301 c	Sorghum	pigeonpea	226 d	251 d	476 d
Limited	wheat	fababean	560 a	500 d	1060 a	Sorghum	pearl millet	225 d	−200 d	25 e
Limited	wheat	rapeseed	280 c	320 e	600 b	Sorghum	mung bean	450 c	−675 f	−225 g
Limited	barley	fababean	480 b	−500 j	−20 d	Pearl millet	pigeonpea	200 d	750 a	950 c
Limited	barley	rapeseed	320 c	640 c	960 a	Pearl millet	mung bean	400 c	−450 e	−50 f
Limited	fababean	rapeseed	−500 hi	320 e	−180 e	Pigeonpea	mung bean	1000 a	450 b	1450 a
**Intercropping means**										
	wheat	barley	−140 d	0.3 e	−140 c	Sorghum	pigeonpea	338 c	250 b	588 b
	wheat	fababean	280 a	750 a	1030 a	Sorghum	pearl millet	338 c	0.1 d	338 c
	wheat	rapeseed	70 b	480 b	550 b	Sorghum	mung bean	675 b	−675 e	0.002 e
	barley	fababean	240 a	−1250 d	−1010	Pearl millet	pigeonpea	300 c	750 a	1050 a
	barley	rapeseed	−0.4 c	480 b	480 b	Pearl millet	mung bean	600 b	−563 e	37 d
	fababean	rapeseed	−750 e	160 c	−590 d	Pigeonpea	mung bean	875 a	112 c	987 a
**Irrigation means**										
Full irrigation				−313 b	−33 b	−346 b	Full irrigation	624 a	−62 b	562 a
Limited irrigation				213 a	240 a	453 a	Limited irrigation	416 b	20 a	437 b
LSD _(0.05)_ for intercropping				55	65	70	LSD _(0.05)_ for intercropping	125	23	134
LSD _(0.05)_ for irrigation				51	59	56	LSD _(0.05)_ for irrigation	176	21	110
LSD _(0.05)_ for irrigation × intercropping				85	94	110	LSD _(0.05)_ for irrigation × intercropping	195	58	210

### Monetary advantage index (MER)

MER is one of the economic profitability indices which is used to identify the profitability or productivity of intercropping system over mono cropping (Table 13). MERs of the data showed that wheat intercropped with fababean had highest economic return followed by wheat intercropped with rapeseed while the rest of the system had a disadvantages of the intercropping as decreased of IA under full irrigated regimes, under limited irrigated regimes all the intercropping system have positive value for which mean all of the had intercropping advantages with highest value for MERs was recorded an intercropping wheat with fababean followed by rapeseed, while lowest value was recorded for MERs in case of barley intercropped with fababean under full irrigated condition. On the other hand, intercropping system of summers crops showed positive value for MERs in all intercropping system except pearl millet intercropped with mungbean under limited irrigated regimes.

**Table 13:** Effect of irrigation regimes on monetary advantages index in different intercropping systems.

Irrigation	Monetary advantages index					
	Winter Season			Summer Season		
	Main crop	Intercrop	System	Main crop	Intercrop	System
Full	wheat	barley	−11144 j	Sorghum	pigeonpea	8846 c
Full	wheat	fababean	28522 a	Sorghum	pearl millet	14923 a
Full	wheat	rapeseed	2877 g	Sorghum	mungbean	8914 c
Full	barley	fababean	−25634	Pearl millet	pigeonpea	12249 b
Full	barley	rapeseed	−2865 i	Pearl millet	mungbean	3133 f
Full	fababean	rapeseed	−11885 j	Pigeonpea	mungbean	7745 d
Limited	wheat	barley	5174 f	Sorghum	pigeonpea	580 i
Limited	wheat	fababean	46456 b	Sorghum	pearl millet	7670 d
Limited	wheat	rapeseed	10160 c	Sorghum	mungbean	−469 j
Limited	barley	fababean	9936 d	Pearl millet	pigeonpea	5638 e
Limited	barley	rapeseed	7562 e	Pearl millet	mungbean	−1123 k
Limited	fababean	rapeseed	663 h	Pigeonpea	mungbean	11821 b
**Intercropping means**						
	wheat	barley	−2985 d	Sorghum	pigeonpea	4713 d
	wheat	fababean	37489 a	Sorghum	pearl millet	11297 a
	wheat	rapeseed	6518 b	Sorghum	mungbean	4222 d
	barley	fababean	−7849 f	Pearl millet	pigeonpea	8944 c
	barley	rapeseed	2349 c	Pearl millet	mungbean	1005 e
	fababean	rapeseed	−5611 e	Pigeonpea	mungbean	9783 b
**Irrigation means**						
Full irrigation			−3354 b	Full irrigation		9301 a
Limited irrigation			13325 a	Limited irrigation		4019 b
LSD _(0.05)_ for intercropping			565	LSD _(0.05)_ for intercropping		530
LSD _(0.05)_ for irrigation			730	LSD _(0.05)_ for irrigation		1050
LSD _(0.05)_ for irrigation x intercropping			750	LSD _(0.05)_ for irrigation x intercropping		840

### System productivity index (SPI)

SPI data of both winter and summer crops presented in Table 14. The data showed intercropping system of cereal with legumes showed that highest SPI under both winter and summer season regardless of water regimes. In case of winter crops highest SPI was recorded in intercropping system of wheat and fababean followed wheat and rapeseed. Barley intercropped with fababean or with rapeseed showed least SPI. In case of summer crops, highest SPI was calculated when sorghum intercropped with pigeonpea followed by pearl millet intercropped with pigeonpea. Data in Table 14 showed that pigeonpea is more productive and can be more successfully grown as compared with mungbean in sorghum or pearl millet intercropping system.

**Table 14:** Effect of irrigation regimes on system productivity index in different intercropping systems.

Irrigation	System productivity index					
	Winter Season			Summer Season		
	Main crop	Intercrop	System	Main Crop	Intercrop	System
Full	wheat	barley	2639 c	Sorghum	pigeonpea	1914 a
Full	wheat	fababean	3684 a	Sorghum	pearl millet	1747 ab
Full	wheat	rapeseed	3450 ab	Sorghum	mungbean	1487 bc
Full	barley	fababean	1231 e	Pearl millet	pigeonpea	1811 ab
Full	barley	rapeseed	1471 d	Pearl millet	mungbean	1698 b
Full	fababean	rapeseed	3147 b	Pigeonpea	mungbean	1576 bc
Limited	wheat	barley	2273 d	Sorghum	pigeonpea	1384 c
Limited	wheat	fababean	2641 c	Sorghum	pearl millet	1152 c
Limited	wheat	rapeseed	2419 cd	Sorghum	mungbean	1255 c
Limited	barley	fababean	1094 e	Pearl millet	pigeonpea	1241 c
Limited	barley	rapeseed	1186 e	Pearl millet	mungbean	1196 c
Limited	fababean	rapeseed	2160 c	Pigeonpea	mungbean	948 d
**Intercropping means**						
	wheat	barley	2456 d	Sorghum	pigeonpea	1649 a
	wheat	fababean	3163 a	Sorghum	pearl millet	1450 b
	wheat	rapeseed	2934 b	Sorghum	mungbean	1371 b
	barley	fababean	1163 e	Pearl millet	pigeonpea	1526 ab
	barley	rapeseed	1329 e	Pearl millet	mungbean	1447 b
	fababean	rapeseed	2653 c	Pigeonpea	mungbean	1262 c
**Irrigation means**						
Full irrigation			2603 a	Full irrigation		1705 a
Limited irrigation			1962 b	Limited irrigation		1195 b
LSD _(0.05)_ for intercropping			220	LSD _(0.05)_ for intercropping		145
LSD _(0.05)_ for irrigation			540	LSD _(0.05)_ for irrigation		330
LSD _(0.05)_ for irrigation × intercropping			335	LSD _(0.05)_ for irrigation × intercropping		230

## Discussion

LER were high for all intercropping systems than sole cropping under both water regimes. Koocheki, *et al*.^51^, reported that intercropping of corn and beans, gives higher LER as compared to sole corn. In intercropping of wheat and lentil, the maximum LER was achieved in lentil and wheat as mixed cropping system^52^. Shaker-Koohi and Nasrollahzadeh^53^ reported that intercropping of sorghum + mungbean, increased LER than sole cropping. Pigeonpea intercropped with mungbean produced maximum LER in both water regimes, this might be due to conducive environment to each other’s. Pearl millet and pigeonpea intercropping had high LER than sorghum and pigeonpea, it might be due the suppressive and allelopathic effect of the sorghum on pigeonpea due to its high stature. The results are in line with Egbe and Kalu^54^, they reported that under high stature sorghum the growth and performance of the pigeonpea was low which resulted in lower LER. However, all crops combination showed higher LER than monocropping, which showed the supremacy of intercropping over monocropping. Legumes intercropped with legumes or legumes intercropped with cereals produced higher LER than cereal intercropped with cereals, one of the major reasons might be due the nitrogen fixation by legumes. In sorghum + mungbean intercropping the growth of mungbean was reduced significantly while the growth of sorghum was increased tremendously, it might due to the strong root system and high nutrients and water absorption capacity of the sorghum. Similar results were reported by the previous researcher like^36,55,56^ they all reported that in cereal-legumes intercropping system cereals were the dominant and aggressive crops while legumes were the suppressive ones. LER value showed the suitability of the mungbean + pigeonpea and pearl millet + pigeonpea intercropping. Alizadeh, *et al*.^57^ reported that intercropping reduced the weed density; in barley and pea intercropping weed biomass than the sole cropping of pea^24^. Barley was more competitive and aggressive in most planting patterns, which is also supported by the finding of Esmaeili *et al*.^58^. Wheat intercropped with fababean gives higher LER than other intercropping system. Intercropping of wheat with fababean had significant effect soil and environmental resources utilization as result product higher LER^20,59,60^. LER for fababean was lower than 0.5 when intercropped with barley, these results are in line with Dhima *et al*.^21^. They reported that LER of fababean was low in intercropping with barley which that barley taking advantages of intercropping while fababean had disadvantages of intercropping. Wheat intercropped with fababean produced higher LER than intercropped with rapeseed. Similar results were reported by Khatun, *et al*.^61^ in wheat-cowpea intercropping highest LER (1.71) was recorded while lowest (1.46) was recoded in wheat mustard intercropping.

Aggressivity value of the intercropping revealed that companion crop did not compete equally. In both seasons i.e., winter and summer cereal showed more aggressivity over their companions’ crops, while might be due the fact that legumes increased nitrogen nutrition of cereals as a result improved it grain yield. Similar results were reported by Kaci, *et al*.^62^ who reported that intercropping fababean with wheat, increase wheat grain nitrogen contents. Sorghum or pearl millet intercrop with mungbean shown highest positive aggressivity over mungbean, it might be due shorter plant stature of mungbean, which was over shaded by sorghum and pearl millet due to which growth of mungbean was severely suppressed and highest benefits of intercropping was got by sorghum and pearl millet. Similar reported was reported by Salih^63^ who stated that sorghum intercropped with legumes removed higher nitrogen from the legumes and suppressed them. Sorghum/millet intercropped with pigeon showed least positive aggressivity value which mean that pigeonpea was most competitive crop with sorghum and pearl millet, it might be due deep-rooted system and high stature of pigeonpea, which make it stronger competitor than mug bean.

The CR is another tool to find the competitive ability of one crop with a companion crop in intercropping. Higher value of CR revealed strong competition on companion crop, under both, water regimes sorghum shown the highest CR value followed by pearl millet over mungbean, which mean that sorghum and pearl millet were most dominant crops over mungbean i.e. mungbean was less competitive with sorghum and pearl millet grown in intercropping for sharing same soil and environmental resources^64^. Relative crowding coefficient (RCC) plays a vital role in finding the competitive effects and intercropping advantages. Barley showed higher RCC than other studied crops except Rapeseed, it might be due the This capacity may be due to the strong nutrient and water competitiveness associated to barley roots in comparison to those of fababean^65^. Agegnehu, *et al*.^20^ also reported similar result that barely have strong dominances over fababean by decrease 50 kg ha^−1^ of fababean. Under full irrigated condition intercropping of pigeonpea + mungbean and sorghum + pigeonpea was highest RCC value. In intercropping of sorghum and mungbean, sorghum was highest RCC value while mungbean had lowest RCC value, which show that sorghum was more superior to mungbean in both water regimes. These results are in line with Banik, *et al*.^18^ in chickpea-wheat intercropping; Ghosh^19^ groundnut-cereal intercropping and Dhima, *et al*.^21^ in cereal-vetch intercropping, cereals were dominant over legumes.

AYL was also an important tool for accessing advantages or disadvantages of intercropping. The results revealed that all the main crops resulted positive value for AYL, which showed that main crops were in advantages of intercropping. The highest grain yield gain was recorded for pigeonpea when intercropped with mungbean, followed by sorghum and pearl millet intercropped with mungbean, respectively, under both, full irrigated and limited irrigated conditions it might be due the high stature of sorghum and pearl millet which over shed the low stature mungbean and decrease the sunlight penetration^36,56^. Under full irrigated regime all the crops intercropped with sorghum showed disadvantages of intercropping except pigeonpea, while under limited irrigated all the crops intercropped with cereals showed negative value for AYL. The highest grain yield loss was recorded for mungbean when intercropped with sorghum and pearl millet under full irrigated condition. Intercropping of pigeonpea and mungbean resulted positive value of for both, pigeonpea and mungbean under both water regimes. These results are in line with those of Banik, *et al*.^25^ who reported that in intercropping of wheat and chickpea, it observed that the chickpea yield in mixture significantly decreased. Layek, *et al*.^56^ also found that soybean yield losses in intercropping with maize due to direct competition for light, space and nutrients. All intercropping systems showed advantages of intercropping. Highest advantage was recorded for pigeonpea, mungbean intercropping system followed by sorghum, pigeonpea and pearl millet, pigeonpea intercropping system, respectively under full irrigated condition while under limited irrigated condition intercropping of pigeonpea and mungbean resulted higher positive value for AYL followed by pearl millet intercropped with pigeonpea. Under limited water condition rapeseed perform better than fababean it might be due to the strong root system of rapeseed which penetrated the soil deeper and possibly the low leaf canopy which conserved the water.

In comparison, intercropping cereals with legumes showed more sophisticated one than intercropping of cereals with cereals crops. It might be due nutrients availability especially nitrogen, in the process of nitrogen fixation by the legumes and strong nutrients and water absorption capacity of the cereals crops as compared with legumes. Similar result was reported by^66^, they reported that an intercropping of fababean + barley, fababean case can cause in crease of 50% in barely aerial biomass, it might be strong root system of barley as compared with fababean^67^. In case of cereals and legumes intercropping system, cereals get more benefits of intercropping and decrees the growth of the legumes Mouradi, *et al*.^65^ who reported that intercropping of barley with fababean, decrease the stem dry weight, root growth weight of fababean. It this experiment that intercropping of fababean with barley is not profitable for the fababean^68^. Growth and yield parameter show negatively effect in fababean as intercropped with cereals particularly with barley^68^. In this research intercropping of cereals with legumes showed most promising interaction with each other and improved the yield and growth of cereals in tram of CR, A, RCC, ATM, LER, LUE etc, it might be due the nitrogen fixation and phosphorus acquisition due to its capability to fixe atmospheric nitrogen and root exudation which improve P solubilization and ensure P availability in soil^69–71^. Fababean is important crop for intercropping with cereals due to several important characteristics like shade tolerance Nasrullahzadeh, *et al*.^72^, nitrogen fixation Li, *et al*.^73^, and high protein content are the unique characteristics which it more suitable to intercropped with cereals.

Higher grains spike^−1^ was recorded under full irrigated regime than under limited irrigated water regime. These results substantiate the outcome noted by El-Sarag.^74^. Sarwar^75^ indicated steady growth of TGW effectively enhanced with required moisture in association to limited water regime. TGW of wheat is statistically significantly affected by irrigation, intercropping and its interaction. Results are at par with results of Ranawake, *et al*.^76^. Among intercropping system wheat + fababean gave highest grain weight. Grain weight significantly varied by intercropping system^25^.

TGW and number of grains head^−1^ or seeds pod^−1^ of all crops were higher in full irrigated condition than limited irrigated condition. The increase in grains weight and number of grains head^−1^/pod^−1^ might the proper moisture availability in the grain’s formation and grain filling stage, which increase the solubility, uptake and transport of plant nutrients. The present results are similar with finding of Khalili^77^ they reported that grains weight reduced under moisture stress condition. Reduction in grains weight are also in line with results of Robertson, *et al*.^78^ who reported decrease in grains weight under moisture stress in mungbean. TGW of all crops were significantly affected by intercropping except millet. Sorghum produced less TGW when intercropped with mungbean; mungbean produced higher TGW when intercropped with pigeonpea, while pigeonpea produced maximum TGW when intercropped with millet. The decrease in grain weight of sorghum intercropped in mungbean might be due to the reason of high number of grains head^−1^, which lead to inadequate assimilate to all grains as result grains weight decreased. The results are in contrast with those of Kumar and Roberts^79^ who reported that different ratios of intercropping did not significant effect on chickpea seed weight. Cereals produced maximum grains head^−1^ in intercropping with legumes than intercropped in another cereal or grown as sole crop. The possible reason might be less interspecific competition in intercropping with legumes. In contrast pigeonpea produced maximum grains pod^−1^ in intercropped with mungbean or grown as sole than intercropped with cereals. But number of grains pod^−1^ in mungbean was statistically similar in intercropping. It might be due to the varying competition among crops for water, space and soil resource, both sorghum and millet were strong competitor with legume as result it suppressed most of the growth of legumes^80^. Pandita *et al*.^81^ also revealed smaller number of grain pod^−1^ in intercropping than sole crop of legumes. Nasarullahzadeh and Koohi^82^ also reported that grains pod^−1^ in mungbean was not significantly affect by intercropping. Under full irrigation condition all crops produced maximum grain yield than limited irrigation condition. The increase in grain yield under full irrigated condition might be due to the high moisture content in the soil, which increased nutrient availability and update and also plant probably increased rate of photosynthesis and translocation of assimilate from leaves and stem toward grains which resulted given higher grain yield. Similar results were reported by Zerbini and Thomas^83^, and Al-Suhaibani^84^, they all reported that increase in grain yield of crops under no water stress condition than water stress condition. High moisture contents in the soil, maintain and improved the turgidity of the plant cells and growth of the plant. Thus, more water uptake by the plants helped in higher transpiration rate, produced more leaf area, high rate of photosynthesis and translocation of assimilate from source to sinks, as result more TGW, high number of grains head, and finally higher grain yield was produced. Similar advantageous effects of high moisture contents in soil on yield attributes, grain and biological yields and dry matter production of millet were also described by Khippal and Hooda^85^ and Imma and Jose^86^. All crops produced higher grain yield in sole cropping than intercropped, probably due to the greater number of plants per unit area in sole cropping. Similar result were reported by Kumar *et al*.^87^; Sharma *et al*.^88^ and Barod *et al*.^89^, they all reported that in mono-cropping the yield of the crops were high than intercropping due to high planting density. Intercropping of cereal with both legumes produced higher grain yield than intercropped with another cereal. The increase in yield was possible due to the conducive environment, less competition for soil resource and more space, more sunlight, to developed high crop canopy as result plants get more assimilate partition and accumulation occurred. Similar results were reported by Tsubo^90^ also reported similar result that beans did not show strong competition in cereal- legumes intercropping. In contrast legumes intercropped in legumes produced higher yield than intercropped with cereals, it might be due to the shading effect of tall cereals and high competition for above and underground resources. The results are in line with Pal *et al*.^80^, who reported that pigeonpea intercropped with urd-bean produced higher yield than intercropped with sorghum.

## Conclusion

From the results derived that all competition indexes showed that intercropping has considerable superiority to monocropping which accredited to better economics and land use efficiency. Competition indexes like land equivalent ratio, aggressivity, competition ratio, area time equivalent ratio, relative crowding coefficient and land utilization efficiency, system productivity index, values were maximum for wheat intercropped fababean in winter season and sorghum/millet intercropped with pigeonpea which indicating the better intercropping system under both irrigation regimes for the semiarid regions.
